# Deciphering the low abundance microbiota of presumed aseptic hip and knee implants

**DOI:** 10.1371/journal.pone.0257471

**Published:** 2021-09-14

**Authors:** Charles Carr, Hannah Wilcox, Jeremy P. Burton, Sharanya Menon, Kait F. Al, David O’Gorman, Brent A. Lanting, Edward M. Vasarhelyi, Michael Neufeld, Matthew G. Teeter

**Affiliations:** 1 Canadian Centre for Human Microbiome and Probiotic Research, Lawson Health Research Institute, London, Ontario, Canada; 2 Department of Surgery, Schulich School of Medicine & Dentistry, Western University, London, Ontario, Canada; 3 Department of Biochemistry, Schulich School of Medicine & Dentistry, Western University, London, Ontario, Canada; 4 Department of Orthopaedics, Adult Hip and Knee Reconstruction Service, University of British Columbia, Vancouver, British Columbia, Canada; 5 Department of Medical Biophysics, Schulich School of Medicine & Dentistry, Western University, London, Ontario, Canada; University of Minnesota Twin Cities, UNITED STATES

## Abstract

16S rRNA gene sequencing of DNA extracted from clinically uninfected hip and knee implant samples has revealed polymicrobial populations. However, previous studies assessed 16S rRNA gene sequencing as a technique for the diagnosis of periprosthetic joint infections, leaving the microbiota of presumed aseptic hip and knee implants largely unstudied. These communities of microorganisms might play important roles in aspects of host health, such as aseptic loosening. Therefore, this study sought to characterize the bacterial composition of presumed aseptic joint implant microbiota using next generation 16S rRNA gene sequencing, and it evaluated this method for future investigations. 248 samples were collected from implants of 41 patients undergoing total hip or knee arthroplasty revision for presumed aseptic failure. DNA was extracted using two methodologies—one optimized for high throughput and the other for human samples—and amplicons of the V4 region of the 16S rRNA gene were sequenced. Sequencing data were analyzed and compared with ancillary specific PCR and microbiological culture. Computational tools (SourceTracker and decontam) were used to detect and compensate for environmental and processing contaminants. Microbial diversity of patient samples was higher than that of open-air controls and differentially abundant taxa were detected between these conditions, possibly reflecting a true microbiota that is present in clinically uninfected joint implants. However, positive control-associated artifacts and DNA extraction methodology significantly affected sequencing results. As well, sequencing failed to identify *Cutibacterium acnes* in most culture- and PCR-positive samples. These challenges limited characterization of bacteria in presumed aseptic implants, but genera were identified for further investigation. In all, we provide further support for the hypothesis that there is likely a microbiota present in clinically uninfected joint implants, and we show that methods other than 16S rRNA gene sequencing may be ideal for its characterization. This work has illuminated the importance of further study of microbiota of clinically uninfected joint implants with novel molecular and computational tools to further eliminate contaminants and artifacts that arise in low bacterial abundance samples.

## Introduction

Clinical and economic outcomes of primary total hip and knee arthroplasty (THA and TKA) are, in general, very positive [1–3]; however, up to 12% of hip and knee implants require revision within 10 years of implantation [4]. Since it is estimated that more than 1.5 million patients received primary THA or TKA in the United States in 2020 [5], need for THA/TKA revision constitutes a substantial financial burden and detriment to patient health. Furthermore, use of THA and TKA is expected to increase by factors of roughly three and four (relative to 2014), respectively, by 2040, owing to an aging and increasingly overweight population, as well as widespread awareness of the benefits of THA and TKA [5]. Consequent increases in need for THA/TKA revision are also anticipated [6], so the economic and health challenges associated with revision will likely become more severe.

Reasons for revision include arthrofibrosis [7–10], fracture [6–11], instability [7–11], and, most commonly, aseptic loosening [6–11] and periprosthetic joint infection (PJI) [6–11]. PJI arises from implant colonization by pathogens [2]. An emerging hypothesis contends that some cases of so-called “aseptic loosening” are, in fact, undiagnosed PJI-related failures [12]. Supporting this hypothesis, numerous prior studies have identified bacteria in presumed aseptic hip and knee implants requiring revision [13–15]. If some failures ascribed to aseptic loosening are caused by undetected bacteria, it will be necessary to characterize the communities of microorganisms associated with clinically uninfected joint implants. Clarifying associations between microbiota and patient demographics, anatomy, and disease would also be important. In these ways, superior diagnostic and treatment protocols could be developed.

The sequencing of the 16S rRNA gene is a promising approach for investigation of the potential microbiota of clinically uninfected hip and knee implants for two reasons. First, numerous studies have already demonstrated its capacity to detect bacteria in joint implants for the diagnosis of PJI [16–18]. Second, the 16S rRNA gene contains highly conserved primer binding sites and several hypervariable regions that differ considerably between taxa, allowing their identification [19]; consequently, it has been widely used for the characterization of other microbiota [20]. There are, however, several issues that are likely to affect sequencing of presumed aseptic hip and knee implant samples. While the 16S rRNA gene PCR primers are mostly considered universal, allowing DNA amplification from most bacterial types, *C*. *acnes*, a common pathogen associated with PJIs, has particularly inefficient amplification with some primer sets [21]. As well, as the amount of true bacterial DNA in a sample decreases, the proportion of spuriously contaminating DNA amplified and detected by 16S rRNA sequencing increases [22, 23]. Finally, contaminants vary between DNA extraction kits and methods [22], so common extraction methodologies might have differential effects on these sparsely microbially populated samples.

Given the clear value of understanding the potential microbiota of presumed aseptic joint implants, as well as the limitations of 16S rRNA sequencing, this study had two main aims. Primarily, this study sought to characterize the polymicrobial communities associated with presumed aseptic hip and knee implants, if extant. Further, this study also aimed to determine the validity and optimization of 16S rRNA gene sequencing to characterize this bacterial population, by evaluating multiple DNA extraction methodologies and applying downstream computational techniques. Bridging these knowledge gaps will aid in understanding the potential role of bacteria in aseptic loosing, and the development of future diagnostic methods.

## Materials and methods

### Patient enrollment

Between August 2019 and March 2020, patients undergoing THA or TKA revision (partial or total) at University Hospital, London Health Sciences Center, London, Ontario were considered for enrollment in the study. As summarized in S1 Fig, 41 patients (20 THA and 21 TKA) were eligible, according to the following exclusion criteria: (1) unwilling or unable to give informed consent, (2) experiencing known or suspected PJI, (3) using antibiotics for previous PJI, (4) requiring revision for the second stage of a two-stage revision for PJI, and (5) not requiring removal of any implant components. Prospective participants were screened for PJI using symptoms, serum C-reactive protein concentration and erythrocyte sedimentation rate, and, if PJI could not be excluded otherwise, joint synovial fluid aspiration (with diagnosis on the basis of the Musculoskeletal Infection Society definition of PJI [24]). The sample size was determined by the availability of participants and funding constraints, as this was a pilot study to motivate further research and guide power calculations. This study was approved by the Western University Health Science Research Ethics Board (REB #114030).

### Sample collection

Standard infection control measures were utilized, including preoperative weight-adjusted antibiotics (cefazolin, except for patients with an allergy) and nasal decolonization with mupirocin, surgical site disinfection (with 2% chlorhexidine-70% isopropyl alcohol or iodine solutions), vertical laminar air flow, and sterile surgical technique. During surgery, new individual sterile scalpel blades and swabs were used to scrape predetermined areas of the implant that were likely sites of biofilm formation and were minimally disturbed by the surgeon (S2 Fig). Samples collected with scalpels were immediately placed in sterile Eppendorf tubes and swabs were returned to their corresponding sterile tubes. An additional sterile Eppendorf tube was left open in the operating room for the duration of each surgery, serving as an open-air control. Open-air controls and implant samples were stored at -20°C prior to DNA extraction. 3–7 additional intraoperative samples were collected for microbiological culture in aerobic, anaerobic, and extended (14 days) conditions. Demographic and clinical data were also recorded for each participant.

### DNA extraction

#### CTAB extraction

21 samples (including controls) from four individuals were thawed, then DNA was extracted using a previously published protocol [25] with modifications for use with human tissue. Specifically, for patient samples, 200 μL of tissue section or the swab tip was aseptically transferred to a 1.5 mL Eppendorf microcentrifuge tube. Open-air control tubes were washed and vortexed with 1 mL of nuclease-free water, then 200 μL of the wash was added to a 1.5 mL Eppendorf microcentrifuge tube. 800 μL of extraction buffer (0.1 M Tris-HCL pH 8, 10 mM EDTA pH 8, 3.5% CTAB) was added to these tubes, which were incubated at 65°C for one hour. The remainder of the protocol was completed as described previously, except samples were incubated overnight (rather than for 1–2 hours) at -20°C in an equal volume of isopropanol and 2 μL of glycogen, the DNA pellet was washed in 1 mL (not 800 μL) of 70% ethanol, and the final DNA pellet was dissolved in 50 μL of 55–60°C nuclease-free water (omitting the RNase A and both TE buffer addition steps). Extracted DNA was stored at -20°C before amplification and sequencing.

#### PowerSoil extraction

The other 286 samples (including controls) were thawed, then DNA was extracted with a high throughput DNeasy PowerSoil HTP 96 Kit (Qiagen, Toronto, ON) in a sterile biological safety cabinet treated with Ambion® RNase AWAY® Decontamination Solution (Molecular BioProducts Inc., San Diego, CA). Gram-positive and -negative bacteria (*S*. *aureus* Newman and *E*. *coli* DH5α) were used as positive controls. DNA and PCR blanks containing only reagents were included to detect bacterial contamination during extraction. DNA extraction was performed according to the manufacturer protocol. Extracted DNA was stored in 96-well plates at -20°C prior to further processing.

### 16S rRNA gene library preparation

A BioMek® 3000 laboratory Automation Workstation (Beckman-Coulter, Mississauga, ON) was used to prepare samples for PCR. CTAB- and PowerSoil-extracted DNA was thawed, and 2 μL of template DNA was aseptically transferred to 96-well plates containing 10 μL 515f and 806r PCR primers (3.2 μM), which amplify the V4 hypervariable region of the bacterial 16S rRNA gene [26]. Then, 20 μL of Promega GoTaq® Colourless Master Mix (Promega, Maddison, WI) was added to the PCR reaction mixture. The 96-well plates were sealed, and DNA was amplified with an Eppendorf Mastercycler® thermal cycler (Eppendorf, Mississauga, ON). An initial 4 minute 95°C heating step was used to activate the GoTaq® polymerase, after which the samples underwent 25 cycles of 95°C for 1 minute, 52°C for 1 minute, and 72°C for 1 minute. The samples were then cooled to 4°C prior to removal from the thermal cycler and storage at -20°C.

### 16S rRNA sequencing

The amplified DNA was thawed prior to sequencing at the London Regional Genomics Center at Robarts Research Institute in London, Ontario. The amplified DNA was quantified using a Quant-iT™ PicoGreen™ dsDNA Assay Kit (Invitrogen), pooled at equimolar concentrations, and cleaned using QIAquick PCR Purification Kit (Qiagen, Germantown, MD) prior to sequencing. 2 × 260 bp paired-end sequencing was conducted using an Illumina MiSeq (Illumina Inc., San Diego, CA).

### 16S rRNA sequencing data analysis

#### Raw read processing

After demultiplexing, sequencing data were processed with the DADA2 pipeline [27]. Of 10 972 240 input paired reads, 10 380 399 remained after initial quality filtering and 10 137 050 were merged after denoising. These reads were assigned to amplicon sequence variants (ASVs), 128 of which were removed (50 did not conform to the expected amplicon size and 78 were chimeric). The remaining ASVs (accounting for 8 250 337 reads) were assigned taxonomy using the DADA2 naive Bayes classifier and version 138 of the SILVA rRNA database [28]. ASVs that were not assigned to a kingdom or were identified as Eukaryota, Mitochondria, or Chloroplast were then filtered out. Across 307 input samples, 570 ASVs and 8 177 228 reads remained for downstream analysis. Demultiplexed reads are available from the Sequence Read Archive (PRJNA726194).

#### Filtering

ASVs accounting for less than 1% of reads in every sample were removed and ASVs with less than 250 reads in total were filtered out. Only samples with at least 50 filtered reads were retained. The dataset was thus reduced to 299 samples, 38 ASVs, and 8 051 444 reads. The concern of false reads is increased by the positive control-associated artifacts. Many of these reads would not have been removed through standard filtering; so, the SourceTracker algorithm (with ɑ_1_ = 0.001 and ɑ_2_ = 0.01) was used to select the subset of patient samples for which reads predicted to have originated from controls accounted for less than 5% of total reads. Remaining contaminant ASVs were detected (and subsequently removed) by frequency and prevalence using decontam [29] with a significance threshold of *p* < 0.2.

#### Downstream analysis

After rigorous filtering, data were analyzed and visualized using R [30]. In all, the following R packages/algorithms were used: DADA2 [27], ggplot2 [31], ggpubr [32], phyloseq [33], decontam [29], zCompositions [34], ALDEx2 [35], vegan [36], and SourceTracker [37]. Statistical significance was set at *p* < 0.05 and effect size > 1. Input counts, taxonomy, and metadata tables, as well as the processed data underlying all figures, are available at 10.5281/zenodo.5136551. Analysis scripts are available on GitHub (https://github.com/charlie-carr/implant_microbiota).

#### PCR

R was used to randomly select 13 samples with zero reads assigned to the only *Staphylococcus* ASV and 13 samples with more than zero such reads. *Cutibacterium* sp. was identified to a reasonable extent in only two samples. These two samples and 26 random samples with zero *Cutibacterium* sp. reads were selected for PCR. PCR was conducted with 5 μL of 10x PCR buffer, 3 μL of 50 mM MgCl_2_, 2 μL of 20 mg/μL BSA, 0.2 μL of each 100 μM primer, 1 μL of Taq polymerase, 2 μL of 10 μM dNTPs, 2 μL of extracted DNA or nuclease-free water (for reagent-only controls), and 34.6 μL of nuclease-free water per sample. *S*. *aureus* [38] and *C*. *acnes* primer information is available in S1 Table. PCR amplicons were resolved by agarose gel electrophoresis and visualized with an ethidium bromide stain.

## Results

### Participants

**Fig 1:**
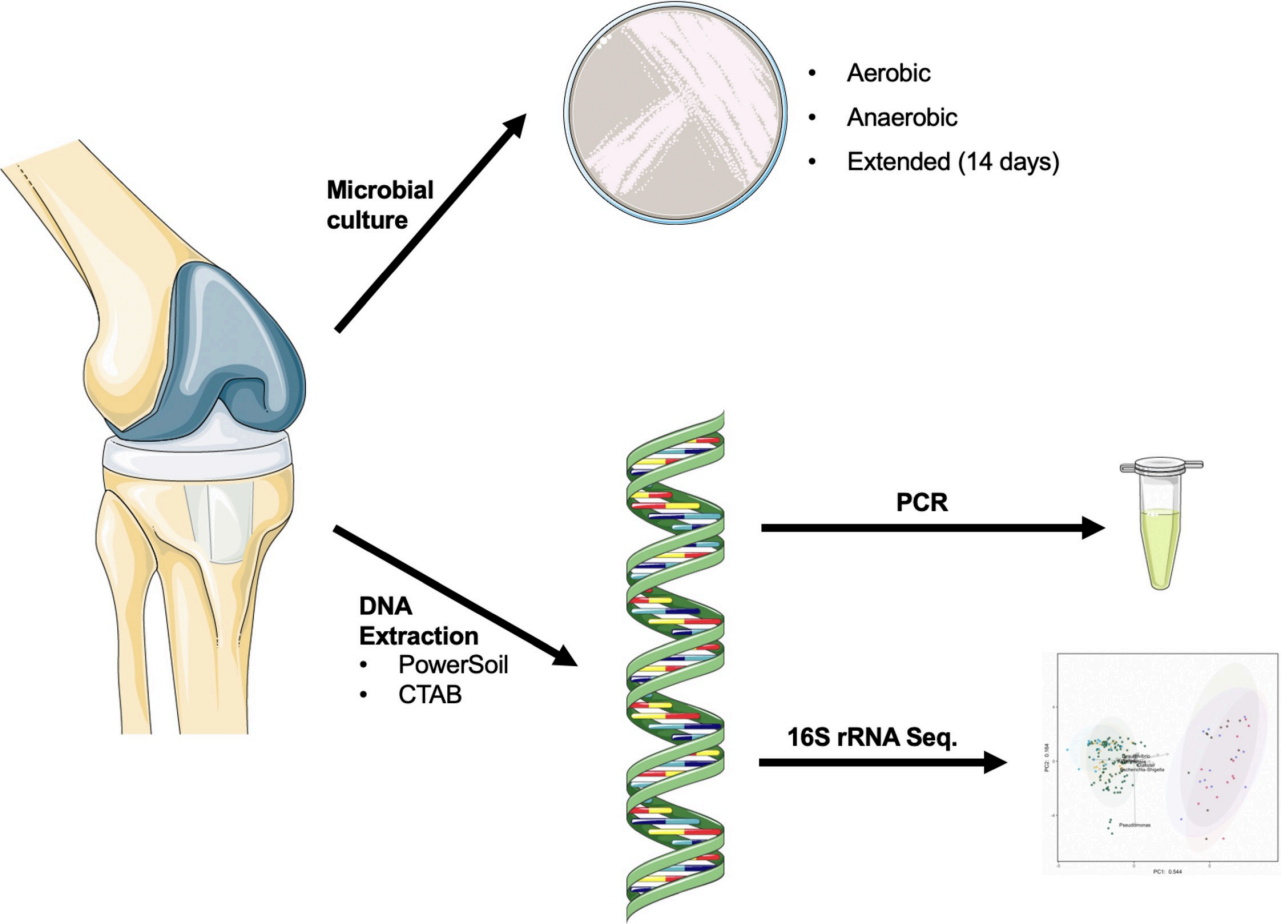
Overview of study design. Samples were collected during revision of presumed aseptic hip and knee implants. Aerobic, anaerobic, and extended microbiological culture was used to diagnosis PJI. DNA was extracted for use in amplification and sequencing of the V4 hypervariable region of the 16S rRNA gene, and for PCR targeted to *C*. *acnes* and *S*. *aureus*. Graphics were obtained from Servier Medical Art by Servier and used under a Creative Commons Attribution 3.0 Unported License.

Samples were collected from 41 patients undergoing THA (*n* = 20) or TKA (*n* = 21) revision. As summarized in Fig 1, samples were collected for routine microbiological culture, as well as DNA extraction for species-specific PCR and sequencing of the V4 hypervariable region of the 16S rRNA gene. Additional patient information is available in Table 1. There were no signs of PJI in any patient in the study at the time of enrollment, but four were subsequently diagnosed with PJI due to unexpected positive intraoperative cultures. These patients were considered in the analysis. Table 2 summarizes the available clinical, microbiological culture, and sequencing data for these patients.

**Table 1:** Study participant information.

Variable		THA and TKA (*n* = 41)	THA (*n* = 20)	TKA (*n* = 21)
**Age in years, median (minimum, interquartile range, maximum)**		70.0 (26, 63.0–74.0, 86)	66.5 (26, 61.5–76.75, 86)	71.0 (56, 66.0–74.0, 84)
**Sex, *n* (%)**	**Female**	23 (56.1)	11 (55.0)	12 (57.1)
	**Male**	18 (43.9)	9 (45.0)	9 (42.9)
**BMI (kg/m** ^**2**^ **), median (minimum, interquartile range, maximum)**		30.1 (23.2, 27.0–35.8, 49.6)	28.3 (23.2, 26.2–30.1, 34.4)	35.8 (23.2, 29.7–40.7, 49.6)
**ASA, *n* (%)**	**2**	8 (19.5)	5 (25.0)	3 (14.3)
	**3**	33 (80.5)	15 (75.0)	18 (85.7)
**Preoperative joint aspirate, *n* (%)**		10 (24.4)	5 (25.0)	5 (23.8)
**Presence of Inflammation, *n* (%)**		1 (2.4)	1 (5.0)	0 (0)
**Preoperative serum CRP ≥ 10 mg/L, *n* (%)**		4 (9.8)	3 (15.0)	1 (4.8)
**Preoperative serum ESR ≥ 30 mm/h, n (%)**		5 (12.2)	3 (15.0)	2 (9.5)
**Etiology of Primary Arthroplasty, *n* (%)**	**Osteoarthritis**	35 (85.4)	15 (75.0)	20 (95.2)
	**Avascular necrosis**	3 (7.3)	2 (10.0)	1 (4.8)
	**Dysplasia**	1 (2.4)	1 (5.0)	N/A
	**Neck of femur fracture**	1 (2.4)	1 (5.0)	N/A
	**Perthes**	1 (2.4)	1 (5.0)	N/A
**Past PJI in Joint, *n* (%)**		1 (2.4)	0 (0)	1 (4.8)
**Prior Revisions, *n* (%)**	**0**	29 (70.7)	16 (80.0)	13 (61.9)
	**1**	8 (19.5)	2 (10.0)	6 (14.6)
	**2**	2 (4.9)	2 (10.0)	0 (0.0)
	**3**	2 (4.9)	0 (0.0)	2 (9.5)
**Etiology of Revision, n (%)**	**Aseptic loosening**	16 (39.0)	8 (40.0)	8 (38.1)
	**Instability**	8 (19.5)	3 (15.0)	5 (23.8)
	**Arthrofibrosis**	5 (12.2)	0 (0)	5 (23.8)
	**Adverse metal reaction**	4 (9.8)	4 (20.0)	0 (0)
	**Polyethylene wear**	4 (9.8)	3 (15.0)	1 (4.8)
	**Chronic patellar dislocation**	1 (2.4)	N/A	1 (4.8)
	**Implant fracture**	1 (2.4)	1 (5.0)	0 (0)
	**Metal allergy**	1 (2.4)	0 (0)	1 (4.8)
	**Pain/mechanical symptoms**	1 (2.4)	1 (5.0)	0 (0)

THA, total hip arthroplasty; TKA, total knee arthroplasty; BMI, body mass index; ASA, American Society of Anesthesiologists Physical Classification System; CRP, C-reactive protein; ESR, erythrocyte sedimentation rate; PJI, periprosthetic joint infection.

**Table 2:** Clinical, microbiological culture, and sequencing data for patients with unexpected positive intraoperative cultures.

	Patient			
Variable	MN7	MN16	MN18	MN29
**Age**	74	62	26	66
**Sex**	Female	Male	Female	Male
**Joint**	Knee	Hip	Hip	Knee
**Type of revision**	One-component	One-component	Modular	One-component
**BMI (kg/m** ^**2**^ **)**	40.7	26.0	33.0	47.3
**ASA**	3	2	3	3
**Preoperative joint aspirate**	N/A	N/A	N/A	N/A
**Preoperative serum CRP (mg/L)**	2.2	4.2	2.9	1.3
**Preoperative serum ESR (mm/h)**	14	7	11	8
**Etiology of Primary Arthroplasty**	Osteoarthritis	Osteoarthritis	Developmental dysplasia	Osteoarthritis
**Past PJI in Joint**	No	No	No	No
**Prior Revisions, n**	0	0	2	0
**Etiology of Revision**	Aseptic loosening	Aseptic loosening	Instability	Aseptic loosening
**UPIC sample**	Tissue	Tissue	Swab	Tissue
**UPIC conditions**	Broth, anaerobic	Broth, aerobic	Broth, anaerobic	Broth, anaerobic
**UPIC results**	*C*. *acnes* and *Anaerococcus octavius*	*Staphylococcus epidermis*	*C*. *acnes*	*C*. *acnes*
**16S rRNA results, % abundance of UPIC bacteria per sample** ^**a**^	0%, 0%, 0%, 0%, 0%, 0%, 0% and N/I^b^	N/A^c^	33.8%, 0%, 57.3%	0%, 0%, 0%, 0%, 1.3%, 0%

BMI, body mass index; ASA, American Society of Anesthesiologists Physical Classification System; CRP, C-reactive protein; ESR, erythrocyte sedimentation rate; PJI, periprosthetic joint infection; UPIC, unexpected positive intraoperative culture; N/A, not applicable; N/I, not identified.

^a^ With 16S rRNA sequencing data collapsed by genera and filtered normally (without SourceTracker or decontam).

^b^*A*. *octavius* was not identified in the filtered 16S rRNA sequencing data.

^c^ The single *Staphylococcus* sp. ASV was altered by the positive control-associated artifact, so the proportional abundance of *Staphylococcus* sp. is inflated in most samples; therefore, it is not appropriate to consider *Staphylococcus* sp. representative of *S*. *epidermis* abundance.

### A positive control-associated artifact is highly significant

**Fig 2:**
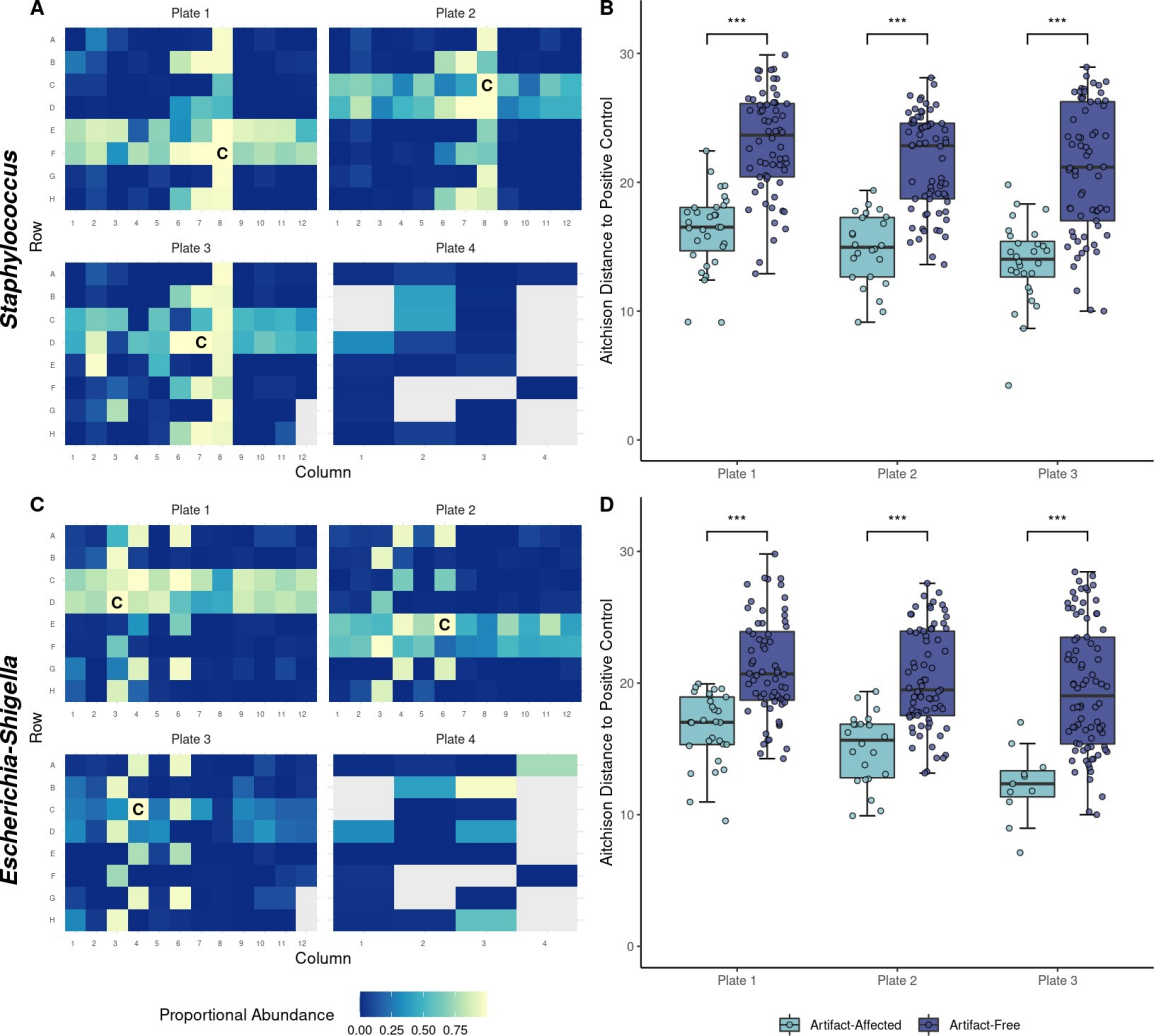
Significant positive control-associated artifacts affect most samples. (A-D) Data was filtered normally for ASVs (without decontam) but not samples since all samples provide insight into the patterns. (A and C) Proportional abundance data from 16S rRNA gene sequencing are presented for two ASVs (*Staphylococcus* sp. and *Escherichia-Shigella* sp.), where the samples are arranged as they were during preparation for sequencing. Grey cells were not used to sequence samples for the present study. Positive controls are denoted with “C”. (B and D) Aitchison distances between the artifact-affected samples and the positive controls (*n* = 31, 24, and 28 for B and *n* = 29, 22, and 11 for D) and those between other samples and the positive controls (*n* = 64, 71, and 65 for B and *n* = 66, 73, and 82 for D) are significantly different (Mann-Whitney *U* test; *p* = 1.1 × 10^−10^, 1.1 × 10^−9^, and 8.1 × 10^−9^ for B and *p* = 2.1 × 10^−7^, 2.1 × 10^−7^, and 1.0 × 10^−5^ for D). Artifact-affected samples were defined as those with at least 50% positive control ASV reads to prevent visual bias in selection. In each test, samples were compared to the type (i.e., *S*. *aureus* or *E*. *coli*) of positive control with which the affected samples were enriched. Samples were only compared to the positive controls on the same plate since plates were prepared separately. Boxplots represent the median (line in box), first and third quartiles (edges of box), and most extreme values in the range median ± 1.5 × interquartile range (whiskers).

Some samples contained unusually large proportions of *Staphylococcus* sp. and *Escherichia-Shigella* sp. 16S rRNA sequencing often cannot identify species, but the *Staphylococcus* sp. and *Escherichia-Shigella* sp. identified in the affected samples are almost certainly the *Staphylococcus aureus* Newman and *Escherichia coli* DH5α positive controls given their abundance and pattern, as well as the fact that they were the only detectable *Staphylococcus* sp. and *Escherichia-Shigella* sp. ASVs. Examination of the sequencing plate layouts reveals that the affected samples are arranged in two patterns (Fig 2A and 2C). First, they appear in bands of two adjacent rows. Second, they are found in vertical, alternating arrangements that differ between the two controls. Of course, there are other samples with relatively high proportions of these ASVs, but the patterns suggest a systematic issue. PCR revealed that none of a random subset of 26 samples contained *S*. *aureus*, whereas the *S*. *aureus* positive controls did (S1 Raw images), further demonstrating that this pattern does not reflect microbial DNA in the patient samples. Finally, analysis with decontam also indicated that the *Escherichia-Shigella* sp. ASV was likely a contaminant, so it was filtered out before conducting most other analyses.

The effect of this artifact was quantified by comparing the Aitchison distances between the affected samples and the positive controls to the Aitchison distances between the other samples and the positive controls. Highly significant differences were observed for *Staphylococcus* and *Escherichia-Shigella* across plates 1–3 (Fig 2B and 2D). The fourth plate did not include *Staphylococcus* or *Escherichia-Shigella* positive controls, so plate 4 samples were excluded from this analysis.

### DNA extraction methodology influences sequencing results

**Fig 3:**
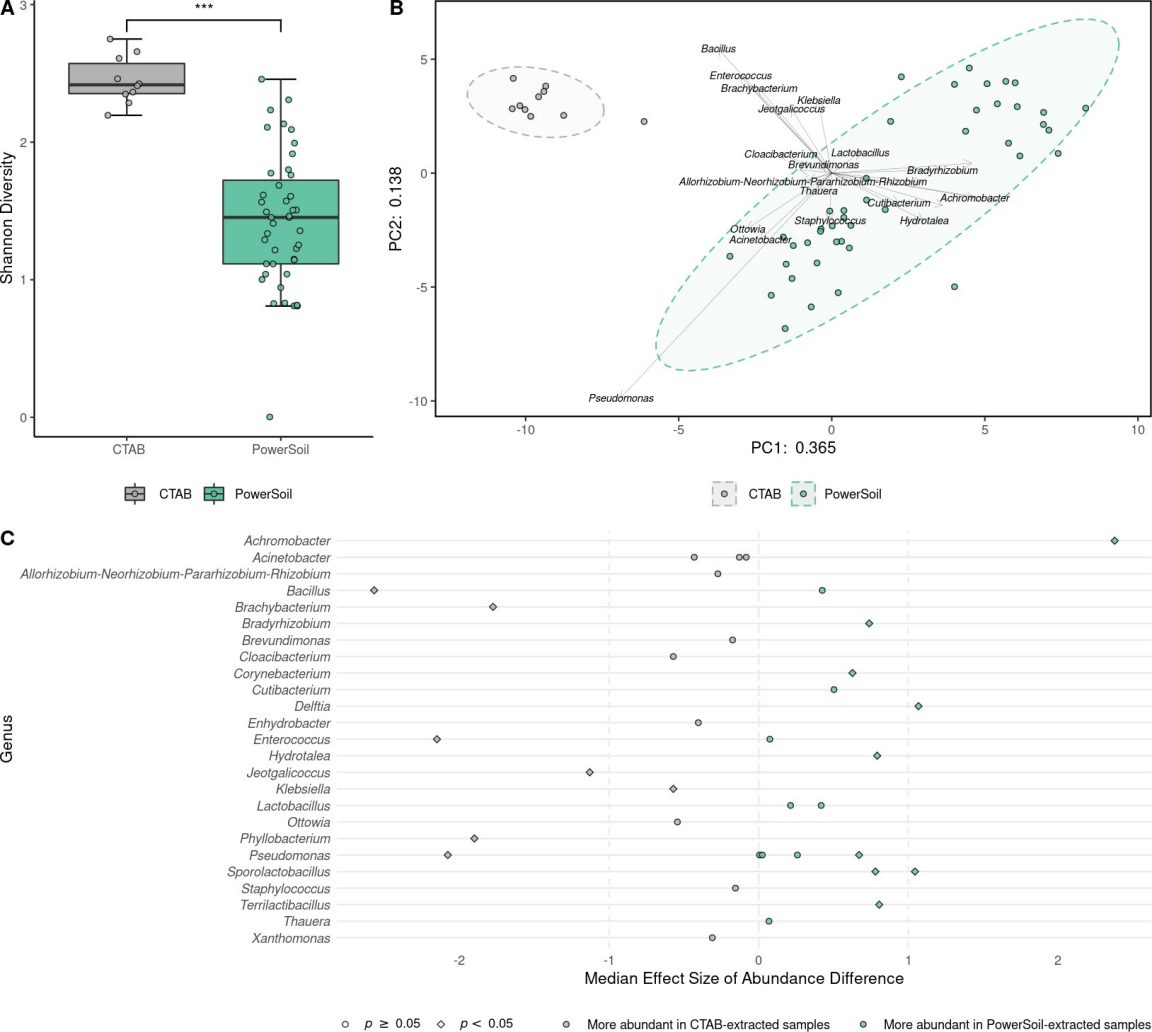
Samples differ significantly in Shannon diversity and abundance of ASVs depending on DNA extraction methodology. Samples were filtered normally and with SourceTracker and ASVs were filtered normally and with decontam. (A) Samples that had DNA extracted using the CTAB methodology (*n* = 10) had significantly higher Shannon diversity than PowerSoil-extracted samples (*n* = 43; Mann-Whitney *U* test; *p* = 9.9 × 10^−9^). Boxplots represent the median (line in box), first and third quartiles (edges of box), and most extreme values in the range median ± 1.5 × interquartile range (whiskers). (B) Principal component analysis of CLR-transformed Aitchison distances (*n* = 10 CTAB-extracted samples and 43 PowerSoil-extracted samples). DNA extraction methodology was significantly associated with the ordination (*envfit* from the vegan package; *p* = 0.001; *r*^2^ = 0.5703). (C) Analysis of differences in ASV abundance between the PowerSoil (*n* = 43) and CTAB (*n* = 10) groups. Effect sizes and *p*-values (Mann-Whitney *U* test with Benjamini-Hochberg correction) were computed with ALDEx2.

Shannon diversity (a measure of ɑ-diversity) was significantly higher in CTAB-extracted samples than PowerSoil-extracted samples (Fig 3A). Fig 3B demonstrates that DNA extraction methodology was also associated with significant differences in overall bacterial composition. ALDEx2 statistical analysis [35] was used to identify differences in the abundances of all ASVs between the CTAB and PowerSoil groups (Fig 3C). On the basis of effect size and *p*-value, *Bacillus* sp., *Brachybacterium* sp., *Enterococcus* sp., *Jeotgalicoccus* sp., *Phyllobacterium* sp., and *Pseudomonas* sp. were significantly more abundant in CTAB samples, whereas *Achromobacter* sp., *Delftia* sp., and *Sporolactobacillus* sp. were significantly more abundant in PowerSoil samples.

### 16S rRNA V4 primers do not robustly amplify *C*. *acnes*

After normal ASV and sample filtering (without SourceTracker or decontam), 22 of 299 samples (7.4%) retained at least one *Cutibacterium* sp. read. However, the proportional abundance of *Cutibacterium* sp. in 20 of those samples was less than 5% (less than 1% in 16 samples), so they would not be considered positive for *Cutibacterium* sp. according to even the most lenient proportional abundance thresholds (one study used 10% [39] and another reported that 59.5% was optimal [40]). The two samples with the highest proportional abundance of *Cutibacterium* sp. (33.8% and 57.3%), which would only be considered positive according to the more lenient threshold, were from one of the four patients with positive intraoperative microbiological cultures. However, cultures from an additional two patients were positive for *Cutibacterium acnes*, whereas 16S rRNA sequencing was negative according to even the 10% criterion.

To properly estimate the frequency of *C*. *acnes* in presumed aseptic hip and knee implants, we used species-specific PCR. The two samples with the highest proportional abundance of *C*. *acnes*, as well as a randomly selected set of 26 samples with zero *C*. *acnes* reads, were analyzed. Both samples with the highest proportional abundance of *C*. *acnes* exhibited amplification, as did at least fifteen of the “negative” samples (S1 Raw images).

### Evidence for clinically uninfected joint implant microbiota

**Fig 4:**
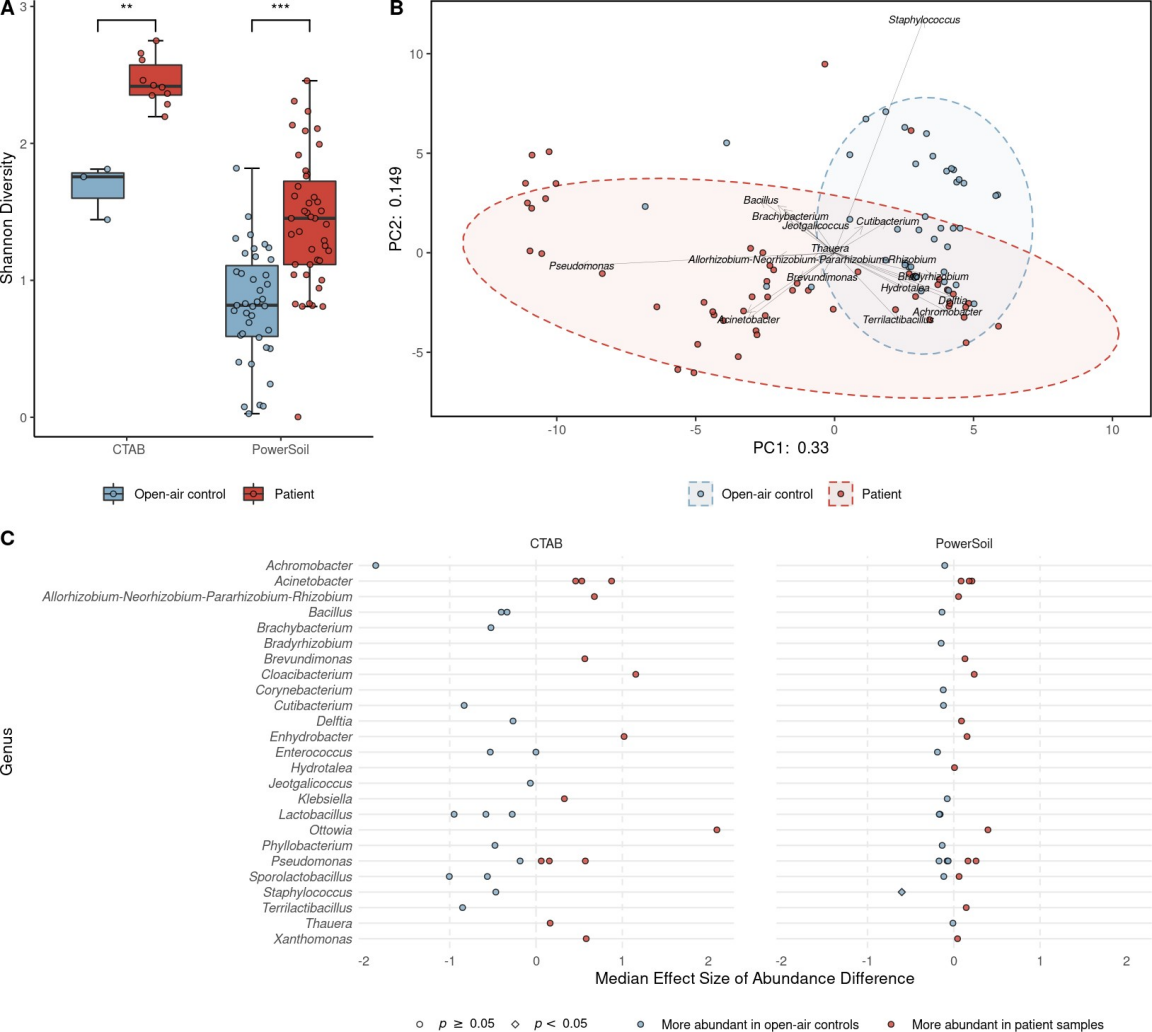
Patient samples contain additional types of bacterial DNA relative to open-air controls, possibly due to their microbiota. Samples were filtered normally and with SourceTracker and ASVs were filtered normally and with decontam. (A and C) Differences between DNA extraction methodologies were controlled for by comparing CTAB samples (*n* = 3 open-air controls and 10 patient samples) and PowerSoil samples (*n* = 39 open-air controls and 43 patient samples) separately. (A) Patient samples had significantly higher Shannon diversity than open-air controls (Mann-Whitney *U* test; *p* = 0.0070 and 4.1 × 10^−8^ for CTAB and PowerSoil groups, respectively). Boxplots represent the median (line in box), first and third quartiles (edges of box), and most extreme values in the range median ± 1.5 × interquartile range (whiskers). (B) Principal component analysis of CLR-transformed Aitchison distances (*n* = 42 open-air controls and 53 patient samples). Sample type (open-air control or patient sample) was significantly associated with the ordination (*envfit* from the vegan package; *p* = 0.001; *r*^2^ = 0.2329). (C) Analysis of differences in ASV abundance between the open-air control and patient sample groups. Effect sizes and *p*-values (Mann-Whitney *U* test with Benjamini-Hochberg correction) were computed with ALDEx2.

After controlling for the previously demonstrated impact of DNA extraction methodology, significant differences were observed in the Shannon diversity of patient samples and open-air controls (Fig 4A). Principal component analysis revealed a significant difference in the bacterial compositions of patient samples versus open-air controls (Fig 4B), and ALDEx2 identified significantly differentially abundant ASVs between patient samples and open-air controls after controlling for DNA extraction methodology. Specifically, the remaining positive control-associated artifact ASV (*Staphylococcus* sp.) and an ASV belonging to a known laboratory contaminant genus (*Achromobacter* [41]) were more abundant in open-air controls. *Cloacibacterium* sp., *Enhydrobacter* sp. and *Ottowia* sp., were all more abundant in patient samples.

### Bacterial features of clinically uninfected joint implants

**Fig 5:**
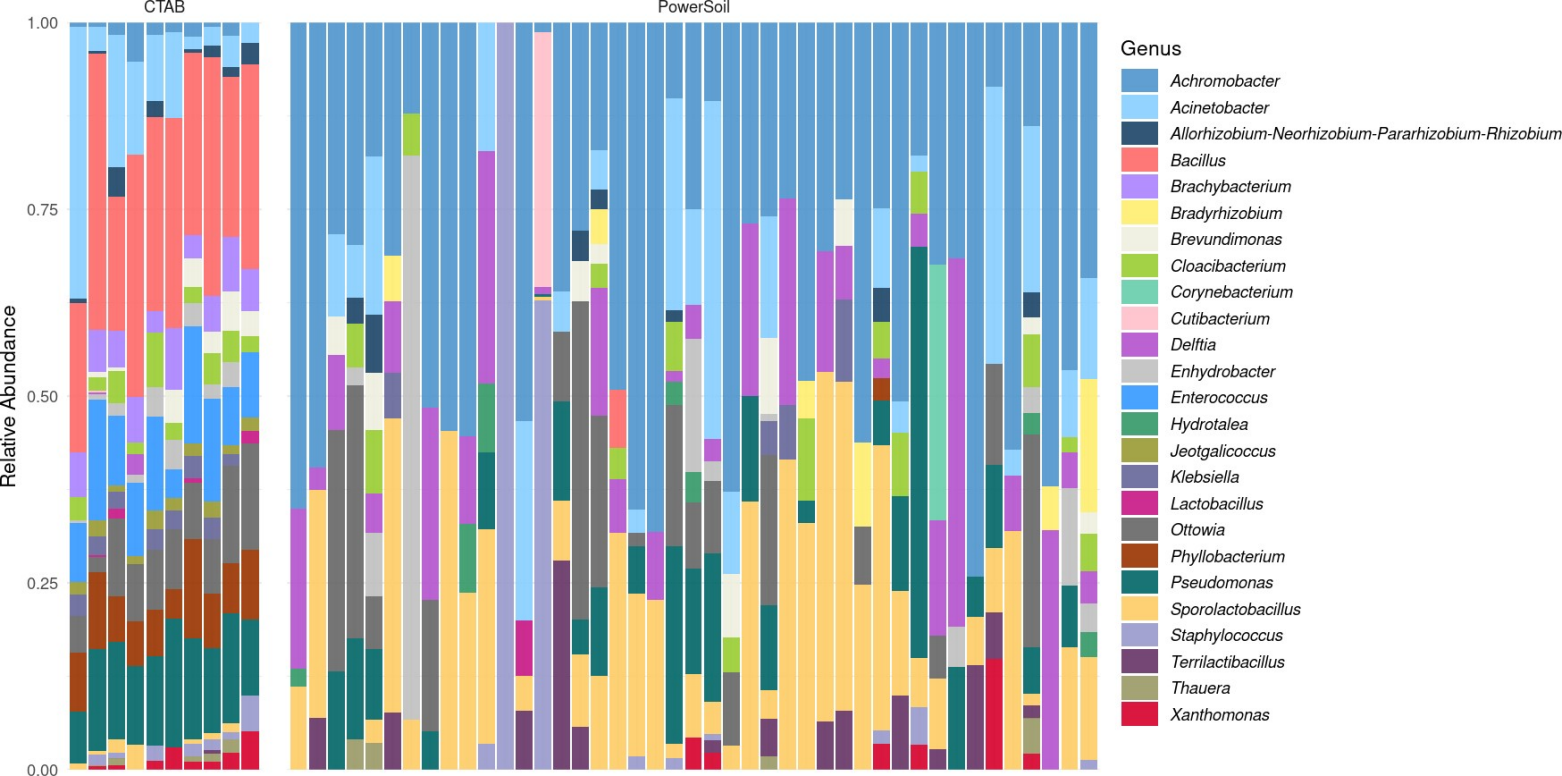
Proportional abundance bar plot of SourceTracker-selected samples. Samples were filtered normally and with SourceTracker and ASVs were filtered normally and with decontam. Each bar represents the bacterial composition of a single sample; bars 1–10 and 11–53 correspond to CTAB- and PowerSoil-extracted samples, respectively. ASVs were collapsed by genus, and the 16S rRNA fractions accounted for by each genus are shown in different colors.

Fig 5 shows bacterial proportional abundance information for the SourceTracker-selected subset of 53 patient samples. Several ASVs—*Acinetobacter* sp., *Ottowia* sp., and *Pseudomonas* spp.—were present at substantial proportional abundances across many samples, regardless of DNA extraction methodology. *Achromobacter* sp. and *Sporolactobacillus* sp. accounted for considerable proportions of the bacterial DNA in many PowerSoil-extracted samples. *Bacillus* sp., *Brachybacterium* sp., *Enterococcus* sp., and *Phyllobacterium* sp. were detected in all CTAB-extracted samples. Note that the PowerSoil-extracted samples with high relative abundance of *Staphylococcus* sp. (*n* = 2) were likely affected by the positive control-associated artifact but not identified as contaminated by SourceTracker.

We leveraged the *isNotContaminant* function of the decontam package—which was used to refute previously published evidence of a low-biomass placental microbiota [29]—to identify likely non-contaminant ASVs. Three *Acinetobacter* sp. ASVs, one *Cloacibacterium* sp. ASV, one *Enhydrobacter* sp. ASV, and one *Ottowia* sp. ASV were identified as non-contaminants. Complete *isNotContaminant* results are available at 10.5281/zenodo.5136551.

Further analysis with *envfit* revealed that the observed polymicrobial communities were patient-specific and were significantly associated with American Society of Anesthesiologists Physical Classification System (ASA) score, revision diagnosis, serum C-reactive protein (CRP) concentration, and serum erythrocyte sedimentation rate (ESR), in addition to DNA extraction methodology. Microbial composition of implant samples did not vary with age, BMI, intraoperative culture results, joint type, primary diagnosis, revision number, sex, or side of the body.

## Discussion

Our findings indicate that the bacterial DNA content in samples derived from presumed aseptic prosthetic knee and hip joints was significantly more diverse than that detected in open-air controls. This finding can be interpreted to support the existence of microbiota in clinically uninfected joint implants. As well, several ASVs (*Ottowia* sp., *Cloacibacterium* sp., and *Enhydrobacter* sp.) were more abundant in implant-derived samples after applying SourceTracker. These data further substantiate the presence of bacteria in presumed aseptic joint implants. We also observed that comparing SourceTracker-selected patient samples to open-air controls resulted in significantly higher abundances of the remaining positive control ASV and a likely contaminant ASV in the open-air controls. These differences support our application of the SourceTracker algorithm because we were successfully able to select patient samples with significantly fewer positive control-associated artifact and contaminant reads.

The differences in Shannon diversity and ASV abundance substantiate previous work that demonstrated the presence of bacteria in presumed aseptic hip and knee implants. One study determined that 22% of samples from knee and hip implants needing revision were culture-positive, whereas immunofluorescence microscopy revealed *C*. *acnes* and/or *Staphylococcus* spp. in 63% of aseptic hip implants [13]. Another investigation used PCR to identify bacterial DNA in 90% of culture-negative hip and knee implants requiring revision for presumed aseptic loosening [14]. Furthermore, one report indicated that 12.1% and 7.9% of presumed aseptic failed hip and knee implants, respectively, were culture-positive [15]. Taking these and other, similar results together, it seems possible that at least some clinically uninfected implants may be colonized by bacteria. However, our findings also support previous work demonstrating the limited utility of universal 16S rRNA PCR-based techniques for the analysis of clinically infected and uninfected hip and knee arthroplasty implants [21, 42, 43].

Specifically, positive control ASVs were enriched in the majority of samples. The precise cause of this issue remains unclear; however, the patterns of effected samples imply an issue related to the PCR primers. Specifically, the bands of two rows on each plate correspond to the two rows that used the same left primer as the positive control. The vertical alternating patterns largely correspond to the wells that used the same right primers as the positive controls. The only exception to this general observation was the presence of the *Staphylococcus* sp. artifact in wells B6, D6, F6, and H6, as the samples in these wells did not share their right primer with a *S*. *aureus* positive control. These inconsistencies contradict the hypothesis that the artifacts were due to a simple demultiplexing error. Furthermore, we did not observe these issues in other microbiota sequencing studies, which relied on the same sequencing instrumentation and demultiplexing script. Most importantly, the artifact was reproduced using Cutadapt [44], an external, widely used tool for the processing and demultiplexing of high-throughput sequencing data (S3 Fig). It is possible that the prevalence of this issue is grossly underestimated because it only manifests so obviously when sparse samples are sequenced with positive controls. To confirm this, future studies could attempt to reproduce this error. This could also facilitate the identification of the source of the error.

In addition, we demonstrated that the unsuitability of V4 16S rRNA primers for the amplification of *C*. *acnes* is highly relevant to 16S rRNA sequencing studies of presumed aseptic hip and knee implants. The low sensitivity to clinically relevant microorganisms like *C*. *acnes* and coagulase-negative *Staphylococcus* has been demonstrated previously and is a significant limitation of universal 16S rRNA primers [21]. Further studies of the microbiota of such implants should include alternative assays for *C*. *acnes* to independently confirm its presence or absence. For example, *C*. *acnes* has successfully been identified using immunofluorescence microscopy [13], culture [15, 21, 45], and RT-PCR [46]. Another promising strategy is a genus-/group-specific real-time PCR panel that targets bacteria typically associated with PJI [47]. The importance of robust methods for *C*. *acnes* detection is increasing as evidence of its presence in presumed aseptic joints grows; for example, a recent report highlighted the presence of *C*. *acnes* in macrophages and stromal cells in shoulder joints requiring primary arthroplasty for osteoarthritis [48].

This study also showed several significant differences between CTAB and PowerSoil DNA extraction methodologies as applied to samples from clinically uninfected hip and knee implants. It is not clear how much of the variation between protocols is due to true discrepancies in capacity to lyse and extract DNA from various taxa or to differences arising from the unavoidable contamination of distinct sets of reagents [22]. Nevertheless, DNA extraction technique was a highly significant confounder, where the CTAB group appeared to have less abundant artifacts and contaminants, when applied to these sparsely microbially populated samples. This result must be considered in the design of 16S rRNA sequencing studies of joint implants and in comparisons between different studies.

The data reported here clearly demonstrate several limitations of 16S rRNA sequencing of samples from clinically uninfected joint implants. Therefore, to study their potential microbiota, further developments in bioinformatics and more targeted methods, such as qPCR, might be required. Future studies may consider use of propidium monoazide-polymerase chain reaction, which prevents amplification of nucleic acids from free or dead microorganisms [49]. To undertake such assays, we must identify taxa of interest. To this end, we provide proportional abundance 16S rRNA gene sequencing data for 53 relatively contaminant-free samples. Our observations of *Brevundimonas* sp. [50], *Cutibacterium* sp. [14], *Lactobacillus* sp. [14], *Pseudomonas* spp. [14], and *Staphylococcus* sp. [14, 50] agree with previously published reports, which demonstrate a microbial presence in samples from presumed aseptic hip and knee implants. Additionally, we identified *C*. *acnes* in patient samples by microbiological culture and species-specific PCR. The concordance between our findings and previous results adds credibility to our application of the SourceTracker algorithm, and to the additional taxa we identify for further investigation: *Acinetobacter* sp., *Cloacibacterium* sp., *Enhydrobacter* sp., and *Ottowia* sp. Supporting these lines of investigation, *Acinetobacter* sp., *Cloacibacterium* sp., *Enhydrobacter* sp., and *Ottowia* sp. were significantly or nearly significantly more abundant in SourceTracker-selected, CTAB-extracted patient samples (effect size of difference in *Acinetobacter* sp. abundance between open-air controls and SourceTracker-selected CTAB samples was 0.90). ASVs assigned to these four genera were all also more abundant (not significantly) in SourceTracker-selected, PowerSoil-extracted patient samples. This consistency, despite the differences in extraction and potential contamination, likely reflects bacteria truly associated with the implants, rather than the same contamination signature across 41 surgeries and two extractions (which are known to be associated with meaningfully different contaminants [22]). Furthermore, decontam *isNotContaminant* results suggest these are non-contaminant ASVs. Importantly, previous work has suggested there might not be consistent differences between these implant-associated polymicrobial communities in cases of diagnosed PJI and presumed aseptic loosening, at least at the coarse level of detail accessible with current sequencing and computational approaches [50]. We observed that there was no significant association between intraoperative culture and 16S rRNA sequencing results, and we detected *C*. *acnes*–a common causative organism of PJI–in presumed aseptic implants, thus providing minimal support for this hypothesis. However, due to the exclusion of PJI cases during patient recruitment, we cannot offer more complete evidence. Also noteworthy is the consistently high proportional abundance of *S*. *maltophilia* in PowerSoil-extracted samples. *S*. *maltophilia* is known to form biofilms [51, 52] and it is therefore tempting to consider it another likely constituent of the microbiota of joint implants. However, analysis with decontam [29] revealed that it is a contaminant (data available at 10.5281/zenodo.5136551). These findings should serve as a valuable guide for future studies of the potential microbiota of clinically uninfected hip and knee implants.

We found that microbial composition of implant samples was associated with some clinically relevant variables (ASA classification, reason for revision, and serum CRP/ESR), but not all (patient age, BMI, joint type, reason for primary joint replacement, revision number, and sex). However, the current study may have been underpowered to detect these associations for secondary outcomes of interest and clinical variables such as reason for primary joint replacement, revision number, BMI, and joint type may play a role in the arthroplasty microbiome.

A limitation of this study was that we compared Shannon diversity and ASV abundances between open-air controls and patient samples to investigate the possible existence of presumed aseptic hip and knee implant microbiota. We considered the incorporation of an environmental control crucial to our study of low bacterial abundance samples [53], and open-air controls were the only feasible control for this analysis. However, at least some of the differences in Shannon diversity and ASV abundance may be explained by unavoidable discrepancies in the bacteria collected in a dry Eppendorf tube versus a tissue sample or swab. Future studies may consider collecting open-air controls in Eppendorf tubes pre-filled with nuclease-free water, similar to the approach taken in a recently published study of the fetal lamb gut microbiome [54]. It is also possible that aseptic failure tissue samples were contaminated after collection. However, the surgeons who collected samples made minimal contact with the implants and did not touch the areas being sampled. In cases where the implant might have been contaminated by contact with another surface, it was not sampled. Therefore, the risk of sample contamination in this study is minimal. Our study was also limited by the inherent challenges of 16S rRNA sequencing. Consequently, *C*. *acnes* would have been underestimated in the analysis of the sequencing results. These issues also likely contributed to the introduction of many contaminant reads, which no filtering paradigm could completely remove, and the highly significant positive control-associated artifacts. Therefore, aggressive filtering parameters, as well as targeted filtering guided by SourceTracker and decontam, were utilized to avoid, as much as was technically possible, the accidental reporting of contaminants. It is not possible to determine which remaining ASVs represent contaminants; however, filtering reduced the number of contaminants identified by decontam from 33 to two and resulted in significantly lower abundance of artifact and likely contaminant ASVs in patient samples (relative to unfiltered open-air controls). So, the influences of contaminants and artifacts on our conclusions are likely small. The application of these filtering procedures decreased the sample sizes used in statistical comparisons, thereby limiting statistical power and reducing our capacity to detect significant relationships. As well, filtering might have removed true features of clinically uninfected hip and knee implants. Finally, the significant differences between bacterial DNA detected in the CTAB- and PowerSoil-extracted groups may reflect an underlying issue for which we have not fully corrected. That said, the taxa we highlight as potentially being associated with presumed aseptic implants were more abundant in patient samples, regardless of extraction methodology, so it is unlikely that this possible issue meaningfully influenced the results because contaminants vary significantly between kits [22]. Given the limitations of sequencing DNA derived from presumed aseptic hip and knee implant samples with 16S rRNA universal primers, we support the further evaluation of specific primers [47], as well as metagenomic shotgun sequencing approaches [55], to investigate microorganisms in presumed aseptic implant failure. Metagenomic shotgun sequencing does not generate the spurious amplicons associated with PCR but is limited by contaminants and the fact that sequence depth is consumed by host DNA [55].

## Conclusion

In summary, presumed aseptic hip and knee implants contain detectable bacterial DNA beyond the background present in open-air controls. This may reflect implant-associated polymicrobial communities, but it may also be at least partially explained by the use of open-air controls in comparisons, contamination, positive control-associated artifacts, and extraction bias between methodologies. Therefore, the data presented here do not confirm a bacterial presence in presumed aseptic implants. They do, however, add to a growing body of evidence supporting the existence of presumed aseptic hip and knee implant microbiota. Further efforts are required to fully decipher the microorganisms associated with clinically uninfected hip and knee implants, as well as the potential role that they may play in aseptic loosening.

## Supporting information

S1 Fig Summary flowchart of patient recruitment process. (TIF)

S2 Fig Sample collection sites on hip and knee implants. (TIF)

S3 Fig The positive control-associated artifact remains after demultiplexing with Cutadapt. Cutadapt, rather than a custom script, was used to process raw reads before DADA2. Otherwise, these heatmaps were prepared as in Fig 2. They show the same clear visual patterns of samples with artificially increased proportional abundance of *Staphylococcus* sp. (A) and *Escherichia-Shigella* sp. (B).
(TIF)

S1 Table Species-specific PCR primer information. (PDF)

S1 Raw images (A and B) Lane 1: DNA ladder. (A) PCR specific for *S*. *aureus* resulted in no amplification in 13 pseudorandomly selected samples with at least one S. aureus read in 16S rRNA sequencing data (lanes 2–14), 13 pseudorandomly selected samples with zero *S*. *aureus* reads (lanes 15–27), and the no-template control (lane 28). There was robust amplification of both *S*. *aureus* positive controls from pure cultures (lanes 29 and 30). (B) Amplification of the three *S*. *aureus* positive controls (lanes 2–4) demonstrates that this lack of amplification is not due to PCR inhibition. Lane 5: no-template control and lane 6: *S*. *aureus* positive control from pure culture. (C) The first set of bands represents the PCR product of interest. Both samples in which *C*. *acnes* was identified by 16S rRNA sequencing showed robust amplification with *C*. *acnes*-specific PCR primers (lanes 19 and 20), like the *C*. *acnes* positive control from pure culture (lane 30). At least 15 samples with no evidence of *C*. *acnes* from 16S rRNA sequencing were positive for *C*. *acnes* according to PCR (lanes 2, 5, 9, 10, 12, 13, 15, 16, 21, 23, 24, 26, 27, 28, 29). This subset includes 3 open-air controls, 1 sequencing negative control, 4 hip samples, and 7 knee samples. Lanes 1 and 32: DNA ladders; lanes 3, 4, 6, 7, 8, 11, 14, 17, 18, 22, 25: samples negative for C. acnes by 16S rRNA sequencing without clear PCR amplification (3 open-air controls, 1 sequencing negative control, 0 hip samples, 7 knee samples); lane 31: no-template control.
(PDF)
