# Supplementary figures and images for: Deciphering the low abundance microbiota of presumed aseptic hip and knee implants
Source: PLoS One. 2021 Sep 14;16(9):e0257471. doi: 10.1371/journal.pone.0257471 (PMC8439452; doi:10.1371/journal.pone.0257471)

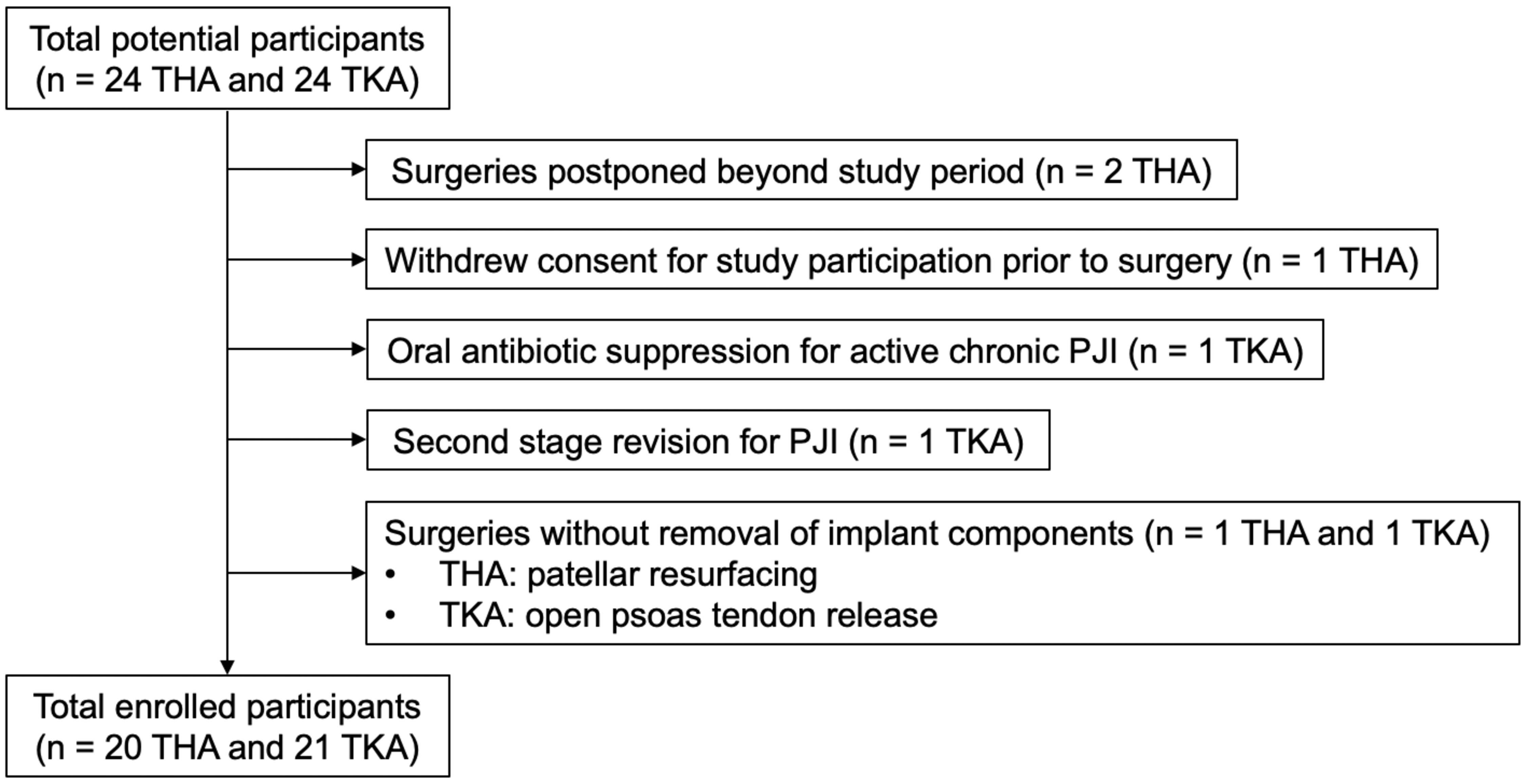

Supplement: S1 Fig — (TIF) [file pone.0257471.s001.tif]

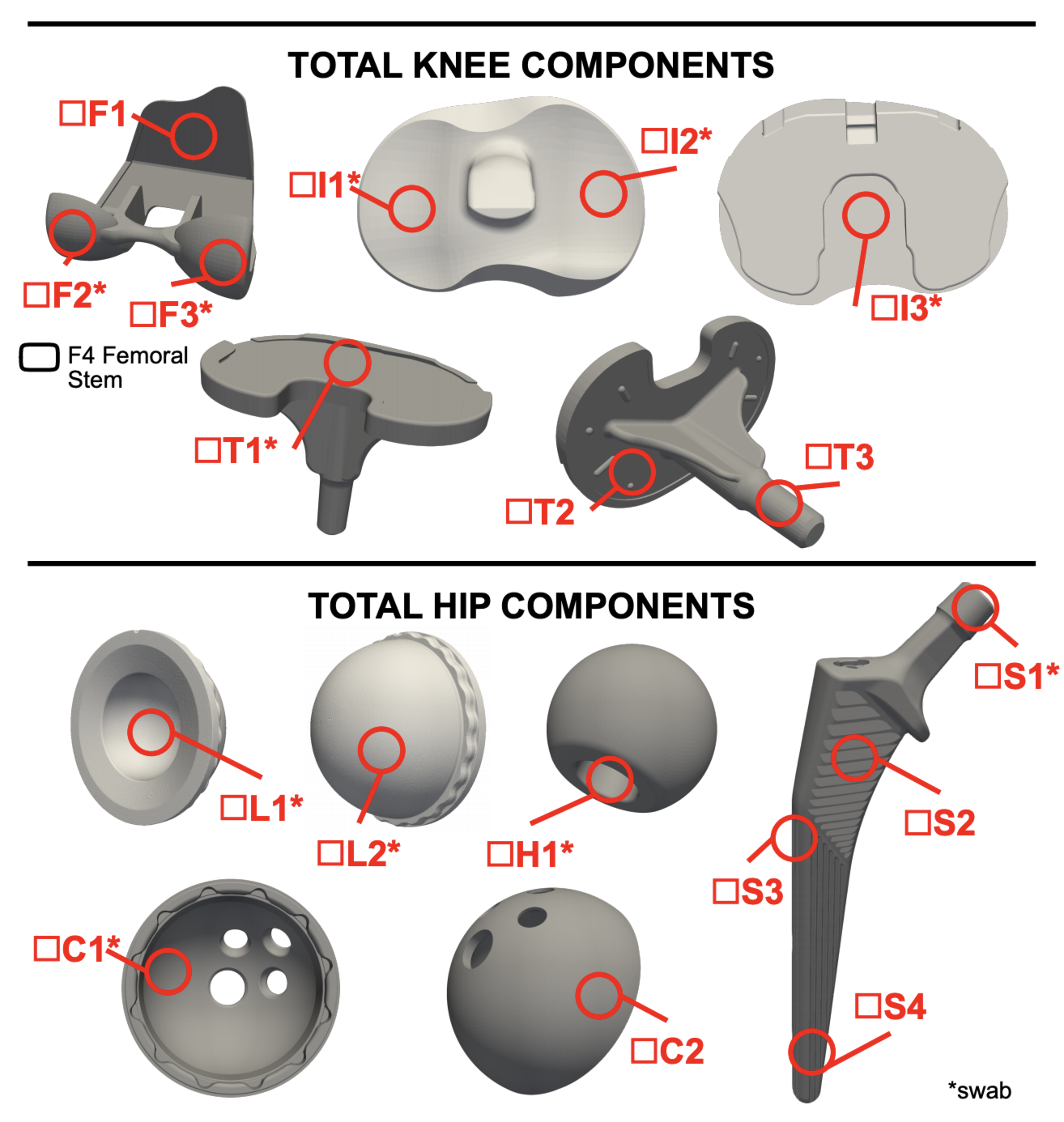

Supplement: S2 Fig — (TIF) [file pone.0257471.s002.tif]

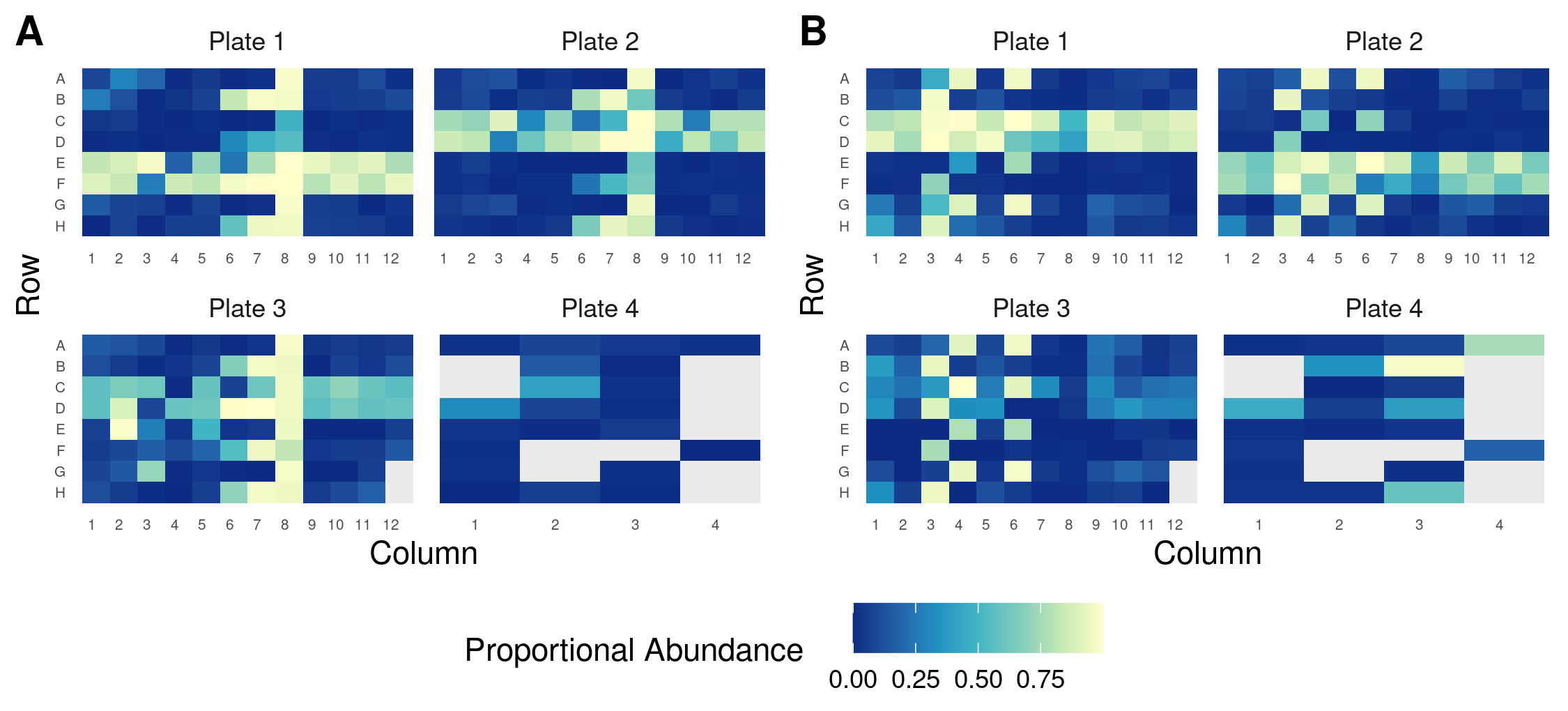

Supplement: S3 Fig — Cutadapt, rather than a custom script, was used to process raw reads before DADA2. Otherwise, these heatmaps were prepared as in Fig 2. They show the same clear visual patterns of samples with artificially increased proportional abundance of Staphylococcus sp. (A) and Escherichia-Shigella sp. (B). (TIF) [file pone.0257471.s003.tif]

A

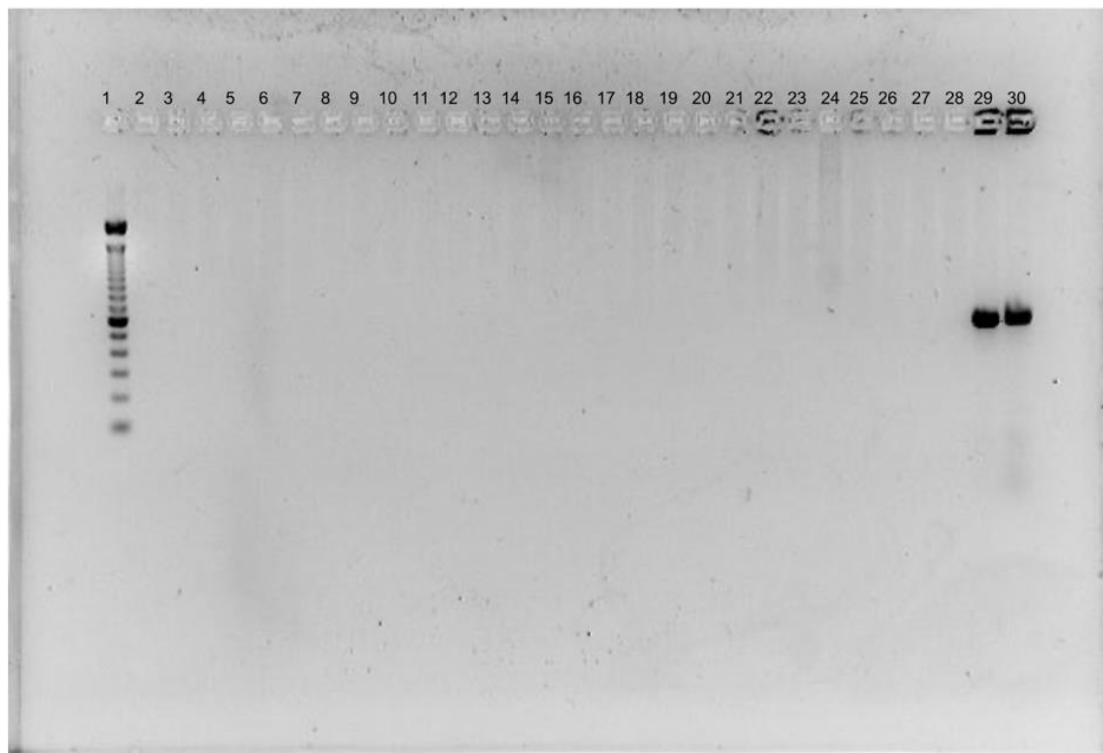

**B**

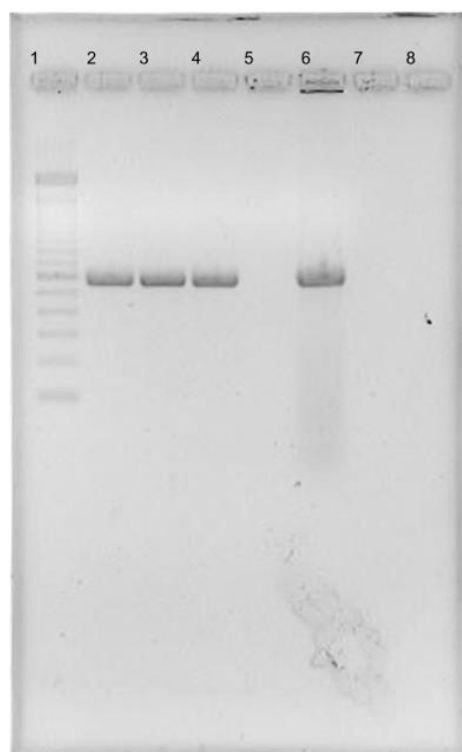

C

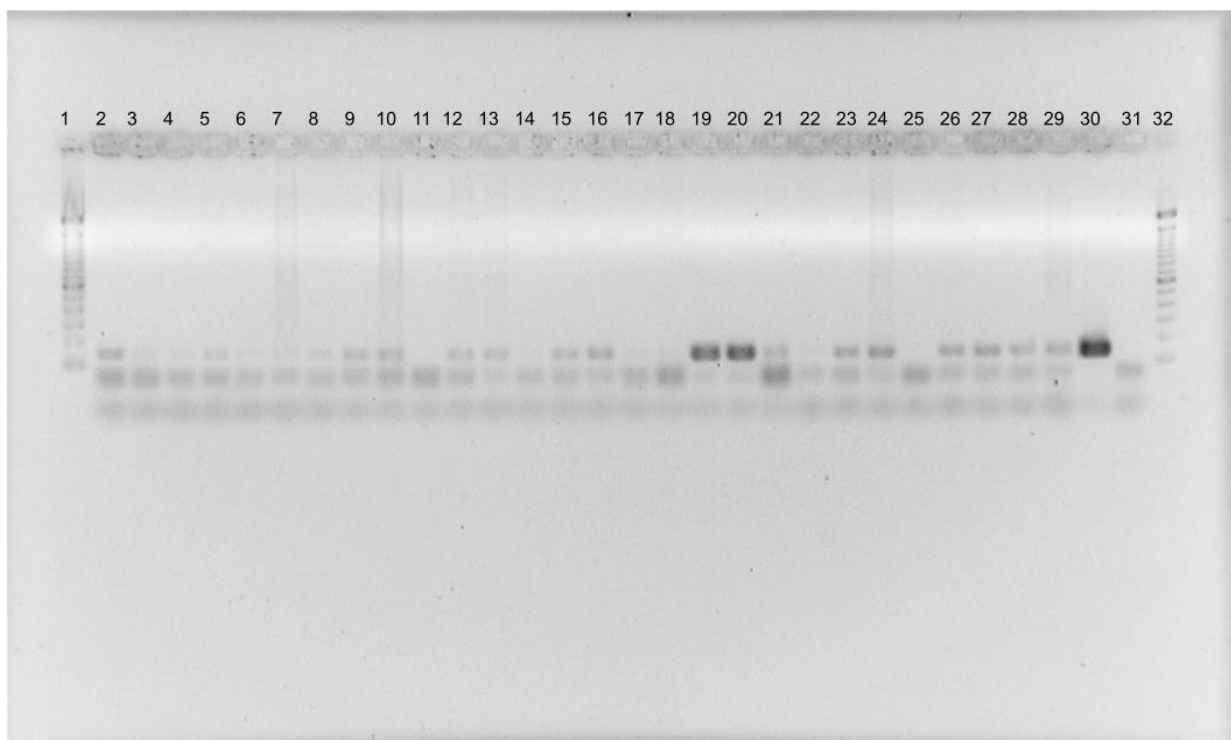

Supplement: S1 Raw images — (A and B) Lane 1: DNA ladder. (A) PCR specific for S. aureus resulted in no amplification in 13 pseudorandomly selected samples with at least one S. aureus read in 16S rRNA sequencing data (lanes 2–14), 13 pseudorandomly selected samples with zero S. aureus reads (lanes 15–27), and the no-template control (lane 28). There was robust amplification of both S. aureus positive controls from pure cultures (lanes 29 and 30). (B) Amplification of the three S. aureus positive controls (lanes 2–4) demonstrates that this lack of amplification is not due to PCR inhibition. Lane 5: no-template control and lane 6: S. aureus positive control from pure culture. (C) The first set of bands represents the PCR product of interest. Both samples in which C. acnes was identified by 16S rRNA sequencing showed robust amplification with C. acnes-specific PCR primers (lanes 19 and 20), like the C. acnes positive control from pure culture (lane 30). At least 15 samples with no evidence of C. acnes from 16S rRNA sequencing were positive for C. acnes according to PCR (lanes 2, 5, 9, 10, 12, 13, 15, 16, 21, 23, 24, 26, 27, 28, 29). This subset includes 3 open-air controls, 1 sequencing negative control, 4 hip samples, and 7 knee samples. Lanes 1 and 32: DNA ladders; lanes 3, 4, 6, 7, 8, 11, 14, 17, 18, 22, 25: samples negative for C. acnes by 16S rRNA sequencing without clear PCR amplification (3 open-air controls, 1 sequencing negative control, 0 hip samples, 7 knee samples); lane 31: no-template control. (PDF) [file pone.0257471.s005.pdf]
