# Supplementary material for: Deciphering the low abundance microbiota of presumed aseptic hip and knee implants
Source: PLoS One. 2021 Sep 14;16(9):e0257471. doi: 10.1371/journal.pone.0257471 (PMC8439452; doi:10.1371/journal.pone.0257471)
Supplement: S1 Table — (PDF) [file pone.0257471.s004.pdf]

S1 Table. PCR Primer Information

**Taxon**	**Target**	**Forward Primer (5' to 3')**	**Reverse Primer (5' to 3')**	**Thermocycler protocol**	**Ref.**
S. aureus	*Sa0836*	GGCGCTTGTAAAATTTTCGT	TGCGCAAAGTTTTATTGAACA	94 °C for 2 min<br>30 cycles of:<br>1. 94 °C for 20 s<br>2. 55 °C for 20 s<br>3. 72 °C for 45 s<br>72 °C for 2 min	34
C. acnes	*CASZ2_13440*	CATGCACAAGTTTGGCTCCC	GAAGAGGTGTGGGTGTCTCG	95 °C for 3 min<br>35 cycles of:<br>1. 95 °C for 1 min<br>2. 60 °C for 30 s<br>3. 72 °C for 45 s<br>72 °C for 10 min	---
